# Upadacitinib unmasks cutaneous T-cell lymphoma in atopic dermatitis

**DOI:** 10.1016/j.jdcr.2024.07.031

**Published:** 2024-08-17

**Authors:** Toan S. Bui, K. Chaney Turney, J. Margaret Moresi, Risa M. Jampel

**Affiliations:** a Department of Dermatology, University of Maryland School of Medicine, Baltimore, Maryland; b Center for Bioethics, Harvard Medical School, Boston, Massachusetts

**Keywords:** atopic dermatitis, cutaneous T-cell lymphoma, mycosis fungoides, Sézary syndrome, upadacitinib

## Introduction

Atopic dermatitis, characterized by recurrent pruritic erythematous plaques, papules, and vesicles, is in-part mediated by T helper 2 cytokines, including interleukin 4 and interleukin 13. Biologic medications, such as dupilumab, and small molecule Janus kinase (JAK) inhibitors, such as upadacitinib, target these cytokines’ signaling pathways. Multiple reports have acknowledged dupilumab’s potential to unmask cutaneous T-cell lymphoma (CTCL)^1^ in patients initially diagnosed with atopic dermatitis. However, upadacitinib has rarely been linked with the emergence of underlying CTCL.^2^ We present a case of a patient treated with upadacitinib for severe atopic dermatitis whom Sézary syndrome developed.

## Case report

A 52-year-old woman with a history of rheumatoid arthritis treated with abatacept, and long-standing atopic dermatitis sought care for uncontrolled atopic dermatitis involving the trunk and extremities. Previous treatments included topical corticosteroids (triamcinolone and clobetasol ointments), methotrexate, intramuscular steroid, and phototherapy with minimal improvement. On July 27, 2022, 2 initial 4-mm punch biopsies of right supraclavicular and dorsal aspect of the left hand were obtained. The biopsies showed irregular epidermal acanthosis with mild spongiosis, exocytosis of small mononuclear cells, and parakeratosis, consistent with patient’s reported history of atopic dermatitis. Dupilumab was initially considered but because of insurance constraints, upadacitinib was chosen. One month after initiation, November 9, 2022, of upadacitinib 15 mg daily, diffuse exfoliative erythroderma developed. She received an oral prednisone taper as well as doxycycline for widespread secondary impetiginization. On November 9, 2022, 2 additional shave biopsies were performed of right thigh and left forearm—showing psoriasiform epidermal hyperplasia with spongiosis and exocytosis of lymphocytes and a few eosinophils. All biopsies performed in 2022 were rereviewed by our in-house dermatopathologist and showed minimal lymphocytic atypia, and were deemed negative for CTCL.

**Fig 1:**
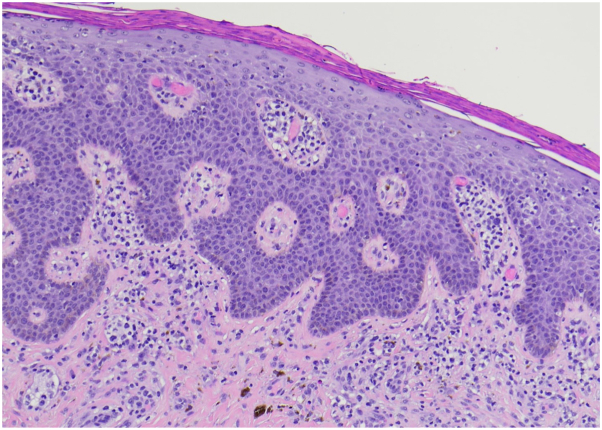
Histologic sections show psoriasiform epidermal hyperplasia with spongiosis and exocytosis of mononuclear cells, some of which are hyperchromatic and slightly enlarged. There is a brisk papillary dermal infiltrate of atypical mononuclear cells in a perivascular and interstitial distribution. The cells are hyperchromatic and enlarged. The intraepidermal atypical cells are CD3^+^, CD8^+^ T cells with a subpopulation of CD4^+^ cells, whereas the dermal cells are predominantly CD4^+^.

The patient’s exfoliative erythroderma recurred after completion of oral steroid taper. Two urgent 4-mm punch biopsies of the back of the right shoulder and upper right back were obtained on February 7, 2023. Dermatopathology findings revealed a mature CD4^+^ T-cell lymphoma with focal Pautrier microabscesses, indicative of CTCL (Fig 1). T-cell gene receptor rearrangement studies were not performed on these skin biopsies. Peripheral blood flow cytometry from the same date showed 1300 cells/μL of neoplastic T cells, with 14% of leukocytes exhibiting CD7^–^ and CD26^–^. Sézary cells were present in the blood. These results confirmed the diagnosis of stage B2 Sézary syndrome/mycosis fungoides, defined as ≥1000/μL of CD4^+^/CD26^–^ or CD4^+^/CD7^–^ cells or other aberrant lymphocyte populations identified by flow cytometry.^3^

**Fig 2:**
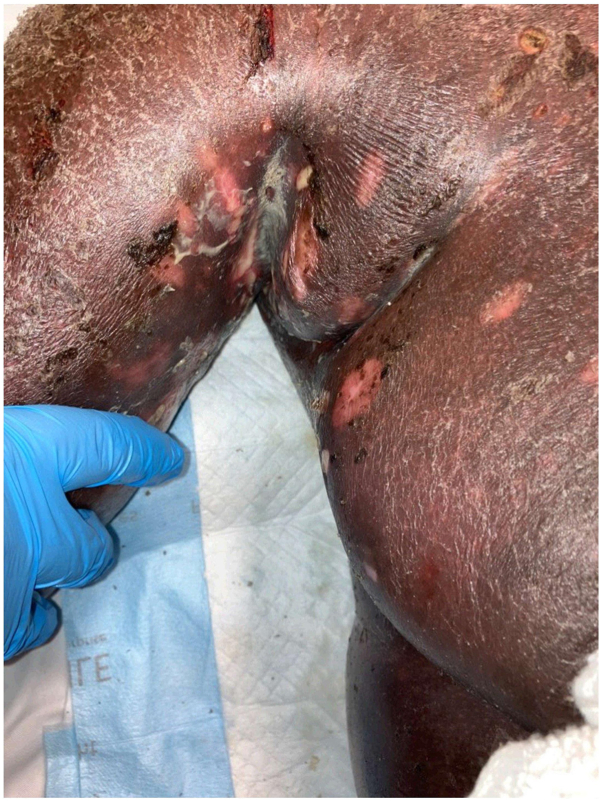
Right axilla with widespread erythematous plaques with scales and scattered erosions.

The patient was promptly admitted, and the medical oncology team started treatment with photopheresis followed by 2 rounds of R-CHOP chemotherapy (rituximab, cyclophosphamide, hydroxydaunorubicin, oncovin, and prednisone). Two repeat 4-mm punch biopsies of inferior upper portion of the arm and superior right arm on May 5, 2023 to assess treatment effectiveness, showed atypical CD4^+^ lymphocytic infiltrate with Pautrier microabscesses—consistent with recalcitrant CTCL. T-cell gene receptor rearrangement studies were not performed on these skin biopsies. Sepsis developed in the patient, most likely because of an impaired skin barrier (Fig 2) in the setting of chemotherapy-induced immunosuppression. She was unable to be treated consistently with fluids and antibiotics because of difficulties with peripherally inserted central catheter line placement. A decision was made by the patient for hospice care. Unfortunately, she passed away on May 17, 2023.

## Discussion

As observed in this case, the diagnosis of CTCL poses challenges because of its oftentimes similar presentation to benign skin conditions, including atopic dermatitis. Dermatologists should be vigilant about considering the diagnosis of CTCL when a patient with treatment-resistant atopic dermatitis and/or presents with sudden-onset erythroderma, especially after the initiation of JAK inhibitors, in this case, upadacitinib. Bloodwork, including peripheral smear and flow cytometry, should be promptly checked when worsening refractory erythrodermic eczema persists despite treatment, especially before considering new biologic medications. Additionally, patients with rheumatoid arthritis have a higher risk of lymphoma, although not specifically CTCL.^4^ Although abatacept is immunosuppressive and exerts effects on T-cell activation, there is currently no literature supporting a direct link between CTCL and abatacept. Likewise, no cases of *de novo* CTCL development have been reported from methotrexate.

In 2021, the Food and Drug Administration issued a warning that JAK inhibitors may be associated with increased risk of lymphomas.^5^ However, the precise mechanism by which upadacitinib either unmasks pre-existing lymphoma or causes malignant transformation is unknown. It has been proposed that JAK inhibitors may compromise antitumor defenses in early-stage mycosis fungoides,^6^ which may be crucial for preventing disease progression to advanced stages. Further research is imperative to uncover and understand the underlying processes driving this phenomenon. Conversely, several case reports and clinical trials have shown a treatment response of CTCL to JAK inhibitors, further highlighting the complex relationship between the 2 entities.^7^
